# Optical conductivity and superconductivity in highly overdoped La_2−*x*_Ca_*x*_CuO_4_ thin films

**DOI:** 10.1073/pnas.2106170118

**Published:** 2021-07-23

**Authors:** Gideok Kim, Ksenia S. Rabinovich, Alexander V. Boris, Alexander N. Yaresko, Y. Eren Suyolcu, Yu-Mi Wu, Peter A. van Aken, Georg Christiani, Gennady Logvenov, Bernhard Keimer

**Affiliations:** ^a^Max-Planck-Institute for Solid State Research, 70569 Stuttgart, Germany;; ^b^Department of Materials Science and Engineering, Cornell University, Ithaca, NY 14853

**Keywords:** high-temperature superconductivity, epitaxial stabilization, phase diagram, optical conductivity

## Abstract

We have used atomic layer-by-layer oxide molecular beam epitaxy to grow epitaxial thin films of ${La}_{2-x}{Ca}_{x}{CuO}_{4}$ with x up to 0.5, greatly exceeding the solubility limit of Ca in bulk systems (x∼0.12). A comparison of the optical conductivity measured by spectroscopic ellipsometry to prior predictions from dynamical mean-field theory demonstrates that the hole concentration p is approximately equal to x. We find superconductivity with $T_{c}$ of 15 to 20 K up to the highest doping levels and attribute the unusual stability of superconductivity in ${La}_{2-x}{Ca}_{x}{CuO}_{4}$ to the nearly identical radii of La and Ca ions, which minimizes the impact of structural disorder. We conclude that careful disorder management can greatly extend the “superconducting dome” in the phase diagram of the cuprates.

## Significance

Chemical substitution is widely used to modify the charge-carrier concentration (“doping”) in complex quantum materials, but the influence of the associated structural disorder on the electronic phase behavior remains poorly understood. We synthesized thin films of the high-temperature superconductor ${La}_{2-x}{Ca}_{x}{CuO}_{4}$ with minimal structural disorder and characterized their doping levels through measurements of the optical conductivity. We find that superconductivity with $T_{c}$ = 15 to 20 K is stable up to much higher doping levels than previously found for analogous compounds with stronger disorder. The results imply that doping-induced disorder is the leading cause of the degradation of superconductivity for large carrier concentration, and they open up a previously inaccessible regime of the phase diagram of high-temperature superconductors to experimental investigation.

The high-temperature superconducting phase in the layered copper oxides is surrounded by Mott-insulating and Fermi-liquid phases for low and high carrier concentrations, respectively, thus generating the “superconducting dome” that has become emblematic for the phase diagram of numerous other quantum materials (1, 2). Whereas the lower end of the dome (for hole concentrations p∼0.05 per Cu atom) is generally ascribed to correlation-driven electron localization, a diverse set of mechanisms has been invoked to explain the disappearance of superconductivity at high doping levels (p∼0.27), without a clear conclusion as to which of these effects is dominant. Recent experimental discoveries on overdoped cuprates have brought the issue into sharp focus. The observation of intense spin fluctuations outside the superconducting dome (3, 4) shows that such fluctuations remain available for Cooper pairing, although spectroscopic experiments indicate that their integrity diminishes (5), and theories predict a reduced coupling strength to fermionic quasiparticles (QPs) (6). The precipitous decline of the superfluid density near the end of the superconducting dome (7, 8) has been controversially discussed in terms of quantum-phase fluctuations (7, 8) and dopant-induced disorder (9–11). The discoveries of charge-density waves (12) and ferromagnetism (13, 14) have highlighted the potential influence of competing instabilities of the electron system, possibly enhanced by a van Hove singularity in the band structure. Very recently, superconductivity has been discovered far outside the superconducting dome established for well-known cuprates (1), for instance, in ${Ba}_{2}$Cu$O_{4-\delta }$ with $T_{c}=73$ K at p∼0.4 (15) and in ${Cu}_{0.75}{Mo}_{0.25}{Sr}_{2}{YCu}_{2}O_{7.54}$ with $T_{c}=84$ K at p∼0.46 (16), suggesting that none of these effects necessarily obliterate superconducting correlations. However, the crystal structure of ${Ba}_{2}$Cu$O_{4-\delta }$ is significantly different from those of other copper oxides (with compressed rather than elongated ${CuO}_{6}$ octahedra), and its electronic structure remains largely unknown. Investigations of other compound families are thus urgently required to assess the implications of this discovery for the theoretical description of high-temperature superconductivity.

**Fig. 1.**
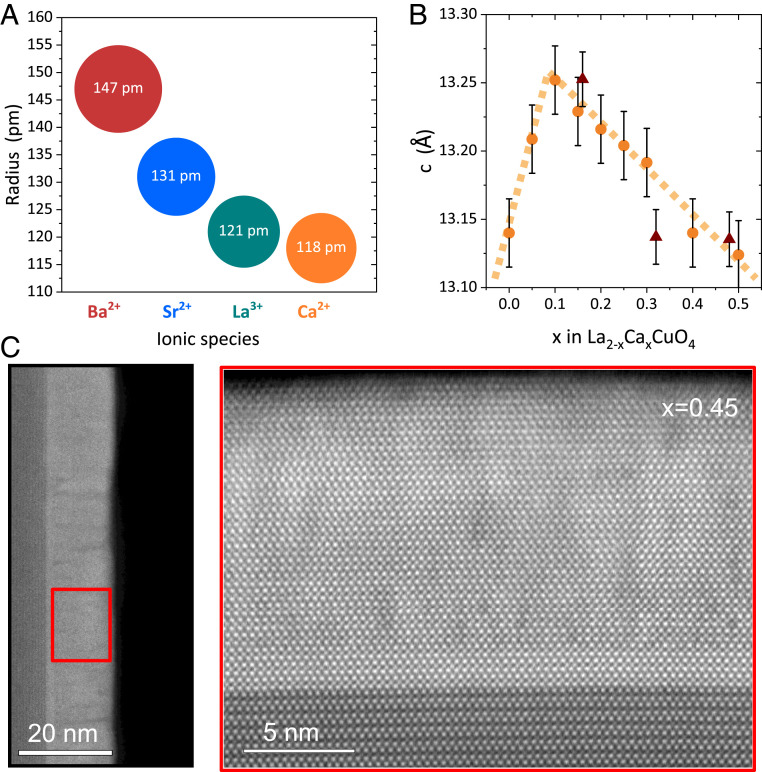
Influence of cation substitution on the lattice structure of 214 compounds. (*A*) Comparison of ionic radii of alkaline-earth cations that are commonly used to substitute ${La}^{3+}$ to realize hole doping. (*B*) Ca concentration dependence of the c-lattice parameter. Circles and triangles indicate thin and thick films, respectively. The dashed lines are guides to the eye. (*C*) STEM images of ${La}_{1.55}{Ca}_{0.45}{CuO}_{4}$. *C*, *Right* shows a magnified view of the marked region in *C*, *Left*.

Here, we report the observation of superconductivity in highly overdoped ${\mathrm{La}}_{2-x}R_{x}{CuO}_{4}$ (R = Ca; LCCO) with x≤0.5. LCCO is a member of the “214” compound family, in which high-temperature superconductivity was first discovered and which remains among the most widely investigated systems due to the simplicity of its cation chemistry and the wide range of obtainable hole concentrations. Unlike ${Ba}_{2}$Cu$O_{4-\delta }$, the Cu coordination and electronic structure of 214 compounds are typical of other cuprates, but as the dopants reside in an atomic layer immediately adjacent to the ${CuO}_{2}$ planes, the electron system is particularly sensitive to structural disorder. For R = Ca, structural disorder is minimal, because its ionic radius is nearly equal to the one of the La ion in the host lattice (Fig. 1*A*). However, the solubility of Ca is limited to x≤0.12 in bulk samples (18, 19), so that overdoped samples could not be investigated.

To overcome this limitation, we have taken advantage of epitaxial stabilization (20), a technique that allows the synthesis of thin films of some complex materials that cannot be prepared in bulk form; prominent examples include the superconducting “infinite-layer” compounds (Ba, Ca)${CuO}_{2}$ (21) and ${Nd}_{0.8}{Sr}_{0.2}{NiO}_{2}$ (22). To determine the doping level of our epitaxially stabilized ${La}_{2-x}{Ca}_{x}{CuO}_{4}$ films, we have measured the optical conductivity using spectroscopic ellipsometry. We find that the optical spectral weight evolves continuously with x up to 0.5, in excellent agreement with predictions of dynamical mean-field theory (17) under the assumption that p≈x. Despite a doping level well outside the widely established superconducting dome (1, 2), we have observed superconducting transitions with $T_{c}=15-20$K in multiple samples. We conclude that structural disorder is a key factor limiting superconductivity in overdoped cuprates and that superconductivity can be stabilized over a greatly extended doping range by careful disorder management. Our findings agree with weak-coupling calculations of the Hubbard model (the simplest generic model of the electron system in the cuprates), which predict a superconducting ground state up to p=1 (23).

Films of thickness 13.2 nm (10 unit cells [u.c.]) and 132 nm (∼100 u.c.) were synthesized by using ozone-assisted atomic layer-by-layer (ALL) molecular beam epitaxy (MBE) on single-crystalline ${LaSrAlO}_{4}$ (001) substrates, at a substrate temperature of 630°C and a pressure of $1\times 10^{-5}$ Torr (more details are in *SI Appendix*, section 1). The growth was monitored by reflection high-energy electron diffraction (RHEED), and the lattice parameters of the completed films were determined by X-ray diffraction. Considering that the in-plane lattice parameters of the tetragonal structure are pinned by the substrate (a=b=3.765 Å), the out-of-plane lattice parameter, c, of LCCO with homogeneously distributed Ca should systematically evolve upon doping in the absence of a structural transition (24). The c parameter of LCCO thin films indeed follows a monotonic doping dependence in 0.15 ≤x≤ 0.5, evidencing the formation of a homogeneous solid solution (Fig. 1*B*). (Note that the nonmonotonic behavior for low x is due to the structural phase transition from orthorhombic to tetragonal lattice symmetry.) In contrast, samples with x≥0.6 exhibit a sharp increase in c and significantly broadened Bragg peaks (*SI Appendix*, section 3).

As a complementary characterization tool of the phase composition and structural integrity of our films, we used atomically resolved scanning transmission electron microscopy (STEM). Specifically, we were interested in the presence of Ca-rich secondary phases, which would lower the Ca content of the LCCO films compared to the nominal value. Whereas the formation of such phases is believed to be the origin of the limited solubility of Ca in bulk LCCO (18), corresponding precipitates are difficult to detect by X-ray diffraction due to their random orientation and the limited scattering power of Ca. Prior work with STEM, on the other hand, has demonstrated that both secondary-phase precipitates and the associated lattice distortions in the matrix metal-oxide films can be readily visualized (25). Large-area surveys of our films showed a highly uniform structure without pronounced secondary-phase precipitates (Fig. 1*C*), in agreement with the evidence for a homogeneous solid solution of Ca in LCCO inferred from X-ray diffraction and Electron Energy Loss Spectroscopy (EELS). Detailed STEM and EELS analyses, along with Rutherford Back Scattering measurements as complementary probes of the Ca content, can be found in *SI Appendix*, section 4.

**Fig. 2.**
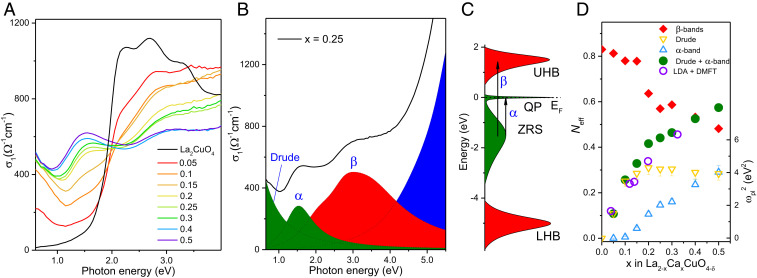
Optical spectra and spectral-weight analysis. (*A*) Doping dependence of the real part of the optical conductivity, $\sigma_{1}\left(\omega \right)$, of thin LCCO films measured at room temperature. (*B*) Decomposition of $\sigma_{1}\left(\omega \right)$ for x=0.25 into separate Lorentzian bands determined by dispersion analysis (*SI Appendix*, section 2). LHB, lower Hubbard band. (*C*) Schematic of the band structure of LCCO adapted from Weber et al. (17). (*D*) Left scale: Doping evolution of the spectral weights of the features labeled in *B*, expressed in terms of the effective number of electrons $N_{eff}$ per Cu atom. The magenta circles represent the spectral weight integrated below 1.5 eV (corresponding to our Drude and α bands) predicted by the six-band LDA + DMFT theory of Weber et al. (17). Right scale: Squared plasma frequency extracted from the Drude peak.

We now present measurements of the optical properties of the LCCO films that are directly sensitive to the doping level. Variable-angle spectroscopic ellipsometry was used to independently obtain the real and imaginary parts of the complex dielectric function ε(ω) = $\varepsilon_{1}\left(\omega \right)$ + i$\varepsilon_{2}\left(\omega \right)$, without the need for Kramers–Kronig transformations. Fig. 2*A* shows that the optical conductivity $\sigma_{1}\left(\omega \right)=\omega \varepsilon_{2}\left(\omega \right)/4\pi$ extracted from these measurements evolves continuously with Ca content over the entire range of compositions from x=0 to 0.5. In the range x=0−0.3, the observed behavior is fully consistent with prior work on ${La}_{2-x}{Sr}_{x}{CuO}_{4}$ (LSCO) (26). Specifically, the optical gap in the x=0 parent compound closes with increasing x, and spectral weight accumulates at low energies, leading to an isosbestic point at 2 eV. The spectral-weight shift evolves smoothly and continuously for x up to 0.5 (Fig. 2*A*). For x≥0.6, on the other hand, the low-energy spectral weight collapses abruptly, in lockstep with the degradation of the structural properties (*SI Appendix*, section 5).

We now proceed to a quantitative description of the optical spectra of doped LCCO, which comprise four distinct features (Fig. 2*B*). Following prior work, these features can be schematically described in terms of an electronic density of states dominated by a band primarily built up of hybridized Cu-$d_{x^{2}-y^{2}}$ and planar O-$p_{x,y}$ orbitals (Fig. 2*C*). Strong electronic correlations generate upper Hubbard bands (UHBs), lower Hubbard bands, and a “Zhang–Rice singlet” (ZRS) band (27) with a narrow QP resonance at the Fermi level. In this picture, the salient features of $\sigma_{1}\left(\omega \right)$ can be assigned to a Drude peak due to free QPs at ω=0; the α-band at ∼1.5 eV due to intra-ZRS transitions (green in Fig. 2*B*); the β-band around 3 eV due to ZRS–UHB transitions (red); and high-energy transitions between various other bands (blue). More comprehensive multiband theories (17) also include electronic states derived from hybridized Cu-$d_{3z^{2}-r^{2}}$ and apical O-2$p_{z}$ orbitals that partially overlap the ZRS states and contribute significantly to the α-band.

To compare our results to theoretical predictions, we fitted the spectra to a superposition of four Lorentzian oscillators corresponding to the features in Fig. 2*B*. The bare plasma frequency $\omega_{pl}$ was determined from the zero-crossing of $\varepsilon_{1}\left(\omega \right)$ in the Drude tail (Fig. 2*D*, right scale). In agreement with prior experimental and theoretical work (17, 26), the x-dependence of $\omega_{pl}$ saturates around optimal doping, so that this quantity is not useful as a means to assess the doping level of our samples. An equally well established—and more instructive—trend is the transfer of spectral weight from the β-band (which reflects transitions with final states in the UHB) to the Drude peak and the α-band (with both initial and final states close to the Fermi level). Simultaneous fits to the independently measured $\sigma_{1}\left(\omega \right)$ and $\epsilon_{1}\left(\omega \right)$ allow for accurate and reliable determination of the spectral weights of both bands. The results are expressed in terms of the dimensionless electron count $N_{eff}$ per Cu atom and plotted in Fig. 2*D* as a function of Ca content. In the range x=0−0.3, the x-dependence of $N_{eff}$ is again fully consistent with prior experimental work on bulk 214 compounds (26). Predictions for the combined spectral weight of the Drude peak and α-band in the framework of a six-band LDA + DMFT model (i.e., density functional theory in the local-density approximation combined with dynamical mean-field theory; magenta circles in Fig. 2*D*) are also in excellent agreement with the experimentally determined $N_{eff}$, under the assumption that p≈x (17) . Remarkably, the trend established for underdoped and weakly overdoped samples continues unabated in the highly overdoped regime, providing confidence that the hole content is indeed approximately equal to x for x≤0.5.

**Fig. 3.**
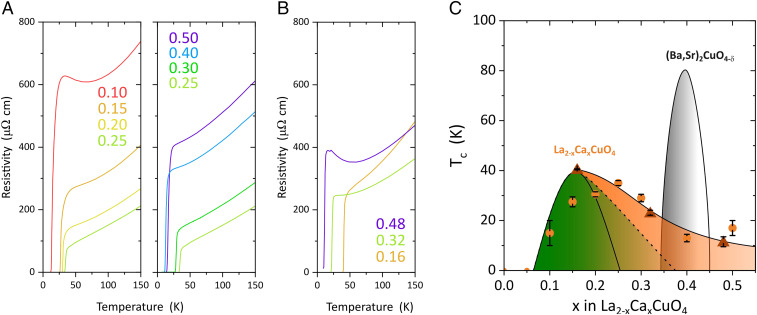
Resistivity and phase diagram. (*A*) Resistivity of LCCO films with thickness $t_{\mathrm{film}}∼13.2$ nm. The numbers in the legend indicate the concentration of dopants. The measurements were performed in van der Pauw geometry with Ag/Au contacts. Note that the residual resistivity may be influenced by a small number of extended defects, such as dislocations nucleated by substrate steps. (*B*) Resistivity of LCCO films with $t_{\mathrm{film}}∼132$ nm. (*C*) Doping dependence of $T_{c}$. Circles and triangles represent $T_{c}$ of films with $t_{\mathrm{film}}=13.2$ nm and ∼132 nm, respectively. $T_{c}$ was estimated as the temperature where the resistivity drops by 50%, and the error bars indicate the widths of the transitions. The green-shaded area indicates the previously established stability range of superconductivity in bulk LSCO (solid line) (1) and LSCO thin films (dashed line) (3, 24, 28). The gray-shaded area corresponds to the phase diagram of (Ba,Sr)_2_Cu$O_{4-\delta }$ (15, 29).

**Fig. 4.**
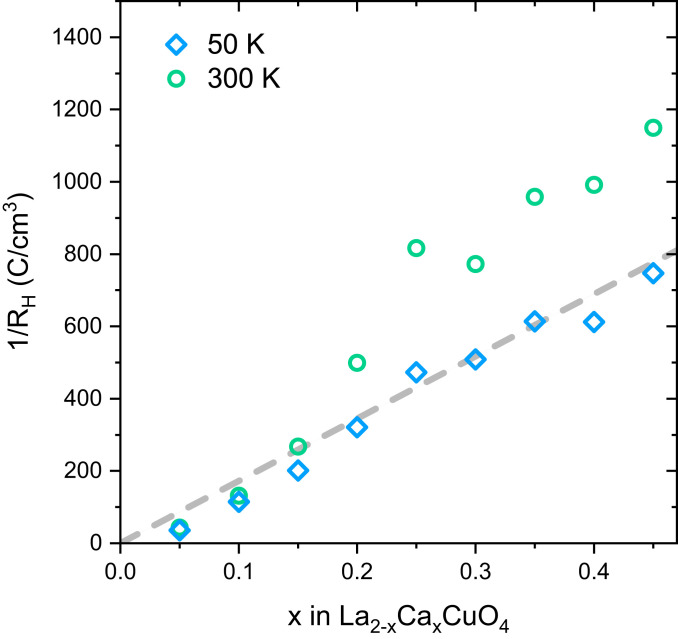
Hall number extracted from Hall-effect measurements in a magnetic field −9 T ≤H≤ 9 T applied perpendicular to the film surface. The Hall effect was found to be linear in H throughout the measured field range. Blue diamonds and green circles correspond to measurements at 50 K and 300 K, respectively. The dashed line is a guide to the eye.

Having established the high homogeneity, crystallinity, and doping level of our LCCO films, we now present their resistivity and superconducting $T_{c}$. Fig. 3 *A* and *B* show the results for thin and bulk-like thick films, respectively. In both sets of samples, the resistivity at 300 K decreases with increasing x up to x = 0.25 and increases for larger x, consistent with prior studies of LSCO (8, 24, 30). Almost all samples with x>0.05 show superconducting transitions with widths below 5 K. For moderate doping, the $T_{c}$ of thin samples is somewhat reduced compared to bulk-like films, possibly due to the enhancement of competing order such as charge-density waves by epitaxial strain (31). The maximum $T_{c}=40$ K observed in the bulk-like LCCO film with x = 0.16 is comparable to the maximal $T_{c}$ in other 214 cuprates. Measurements of the Hall effect (Fig. 4) showed a strong temperature dependence, which is common in the normal state of cuprate superconductors. Nevertheless, for both temperatures, the Hall number increases monotonically with doping, supporting the hypothesis p≈x. Note that pronounced anomalies of the Hall effect due to the doping-induced Lifshitz transition of the Fermi surface topology are only expected in the high-field limit (32).

The key observation of the transport measurements is the persistence of superconductivity with $T_{c}=15-20$ K in highly overdoped films up to x = 0.5, which is much higher than the dopant concentration at which the superconducting dome ends in bulk samples (1) and in epitaxially stabilized MBE-grown LSCO films (3, 24, 28) (solid and dashed lines in Fig. 3*C*, respectively). The fact that both 10-u.c.- and 100-u.c.-thick films exhibit consistent behavior implies that the persistent superconductivity does not originate from the interface between substrates and films, but is intrinsic to LCCO. Following ${Ba}_{2}$Cu$O_{4-\delta }$ (15) and related materials (16, 33), LCCO is thus the second family of cuprates that exhibits superconductivity well beyond the previously established superconducting dome. As the crystal structure of LCCO is typical of other cuprates and we were able to establish the doping level with good accuracy, our observations also suggest that a phase diagram with a superconducting phase extending to at least p=0.5 should be regarded as generic to the cuprates.

Fig. 1*A* contains important information about the mechanisms influencing the stability range of superconductivity in the cuprates. In both LSCO and LCCO, conduction electrons are affected by the random variation of the cation charge induced by the substitution of ${Sr}^{2+}$ or ${Ca}^{2+}$ for ${La}^{3+}$ close to the ${CuO}_{2}$ planes, but this effect should influence both compound families in roughly equal proportion. The extended stability range of superconductivity in LCCO can therefore primarily be attributed to the lower level of structural disorder. Dopant-induced disorder has recently received considerable attention following the discovery of an anomalous suppression of the superfluid density in overdoped LSCO thin films (7, 8), which raised questions about possible noncondensed charge carriers in the superconducting state. A series of studies attributed this behavior to Cooper-pair breaking by cation-induced disorder and predicted that superconductivity could persist in the highly overdoped regime of cuprates with reduced disorder (9–11). Our results confirm this prediction.

The situation is thus analogous to the iron arsenides, where it was found that in compounds doped by chemical substitution outside the electronically active FeAs layers (which minimizes the impact of structural disorder), superconductivity persists over a much wider range of the phase diagram than in compounds that are doped by substituting Fe within the layers (34–36). Like in the Fe-based superconductors, our results on the cuprates are significant for the microscopic understanding of superconductivity. In particular, weak-coupling models of the single-band Hubbard model (the simplest generic model for the electron system in the cuprates) predict a superconducting ground state over a wide range of doping levels up to and including p=1 (*SI Appendix*). Weak-coupling theories are known to be inadequate for small p, where strong correlations generate a host of competing ground states with spin and charge order, but are expected to become more accurate in the Fermi-liquid regime at high doping (2). Our results are consistent with the prediction of persistent superconductivity derived from these calculations and with the notion that large-angle impurity scattering (rather than diminished pairing strength) is the leading mechanism underlying the loss of superconductivity in real materials (37).

The results we have presented establish epitaxially stabilized LCCO as a model system for the highly overdoped regime of the cuprates. It should be instructive to determine the Fermi surface and band dispersions of electrons in this regime and their coupling to collective excitations such as phonons and paramagnons, as well as the symmetry of the superconducting order parameter, which has been predicted to change at high doping levels (*SI Appendix*). Finally, the shape of the superconducting phase boundary in Fig. 3*C* suggests that superconductivity might be stabilized at even higher doping levels if they can be realized with minimal disorder, for instance, by further optimization of the MBE growth conditions, or by taking advantage of interfacial charge transfer in heterostructures (38).

## Materials and Methods

Thin films were grown on ${LaSrAlO}_{4}$ (001) single-crystalline substrates (Crystec GmbH) by using an ozone-assisted ALL-MBE system (DCA Instruments). The growth was monitored by using in situ RHEED. During growth, the substrate temperature was kept at 630°C, according to the radiative pyrometer, and the pressure was $∼1\times 10^{-5}$ Torr. To obtain the accurate composition of the films, the effusion cells were calibrated before every growth run by using a quartz-crystal microbalance. The c lattice parameters were measured by high-resolution X-ray diffraction using a Cu-Kα source and a high-resolution diffractometer (Bruker GmbH). The electric transport measurements were performed with a Physical Property Measurement System (Quantum Design, Inc.) in the van der Pauw geometry, using Ag/Au metallic contacts deposited on the four corners of square-shaped samples.

The ellipsometric measurements were performed at room temperature with a variable-angle spectroscopic ellipsometer (J. A. Woollam Inc.) in the energy range 0.55 to 6.5 eV at different angles of incidence (φ = 65°, 70°, and 75°). The ellipsometric parameters Ψ and Δ are defined by tan $\Psi e^{i\Delta }$ = $r_{p}/r_{s}$, where $r_{p}$ and $r_{s}$ are the complex Fresnel coefficients for light polarized parallel and perpendicular to the plane of incidence, respectively. The real and imaginary parts of the complex dielectric function, ε(ω) = $\varepsilon_{1}\left(\omega \right)$ + i$\varepsilon_{2}\left(\omega \right)$, and the related optical conductivity $\sigma_{1}\left(\omega \right)=\omega \varepsilon_{2}\left(\omega \right)/\left(4\pi \right)$ were directly determined from Ψ(ω) and Δ(ω) (*SI Appendix*, Fig. S1). The ellipsometric data were fitted by point-by-point regression analysis to a film-on-substrate model.

To separate contributions from the different bands, we fitted a set of Drude–Lorentz oscillators simultaneously to $\varepsilon_{1}\left(\omega \right)$ and $\varepsilon_{2}\left(\omega \right)$: $\varepsilon_{1}\left(\omega \right)$ + i$\varepsilon_{2}\left(\omega \right)=\varepsilon_{\infty }-\frac{\omega_{pl}^{2}}{\omega^{2}+i\omega \Gamma_{D}}+\sum_{j}\frac{S_{j}}{\omega_{j}^{2}-\omega^{2}-i\omega \Gamma_{j}}$, where $\omega_{j}$, $\Gamma_{j}$, and $S_{j}$ are the peak energy, width, and oscillator strength of the jth oscillator, respectively; $\omega_{pl}$ and $\Gamma_{D}$ are the unscreened QP plasma frequency and scattering rate; and $\varepsilon_{\infty }$ is the contribution of higher-energy interband transitions to the dielectric permittivity. The spectral weight of separate bands was quantitatively analyzed in terms of the effective number of electrons per Cu atom in a u.c., $N_{eff}^{pl,j}$ = $\frac{2m}{\pi e^{2}N_{Cu}}\frac{\omega_{pl}^{2},S_{j}}{8}$, where m is the free electron mass and $N_{Cu}\approx 1.07\times 10^{22}\;{cm}^{-3}$ is the density of Cu atoms.

## Supplementary Material

Supplementary File

## Data Availability

All data used in this study are included in the article and *SI Appendix*.
