# Supplementary material for: Optical conductivity and superconductivity in highly overdoped La2−xCaxCuO4 thin films
Source: Proc Natl Acad Sci U S A. 2021 Jul 23;118(30):e2106170118. doi: 10.1073/pnas.2106170118 (PMC8325326; doi:10.1073/pnas.2106170118)
Supplement: Supplementary File [file pnas.2106170118.sapp.pdf]

Supplemental Information for Optical conductivity and superconductivity in highly overdoped $La_{2-x}Ca_xCuO_4$ thin films

Gideok Kim,[1] Ksenia S. Rabinovich,[1] Alexander V. Boris,[1] Alexander N. Yaresko,[1] Y. Eren Suyolcu,[2,1] Yu-Mi Wu,[1] Peter A. van Aken,[1] Georg Christiani,[1] Gennady Logvenov,[1] and Bernhard Keimer[1]

[1]*Max-Planck-Institute for Solid State Research, Heisenbergstrasse 1, 70569 Stuttgart, Germany*

[2]*Department of Materials Science and Engineering, Cornell University, Ithaca, NY 14853, USA*

(Dated: June 18, 2021)

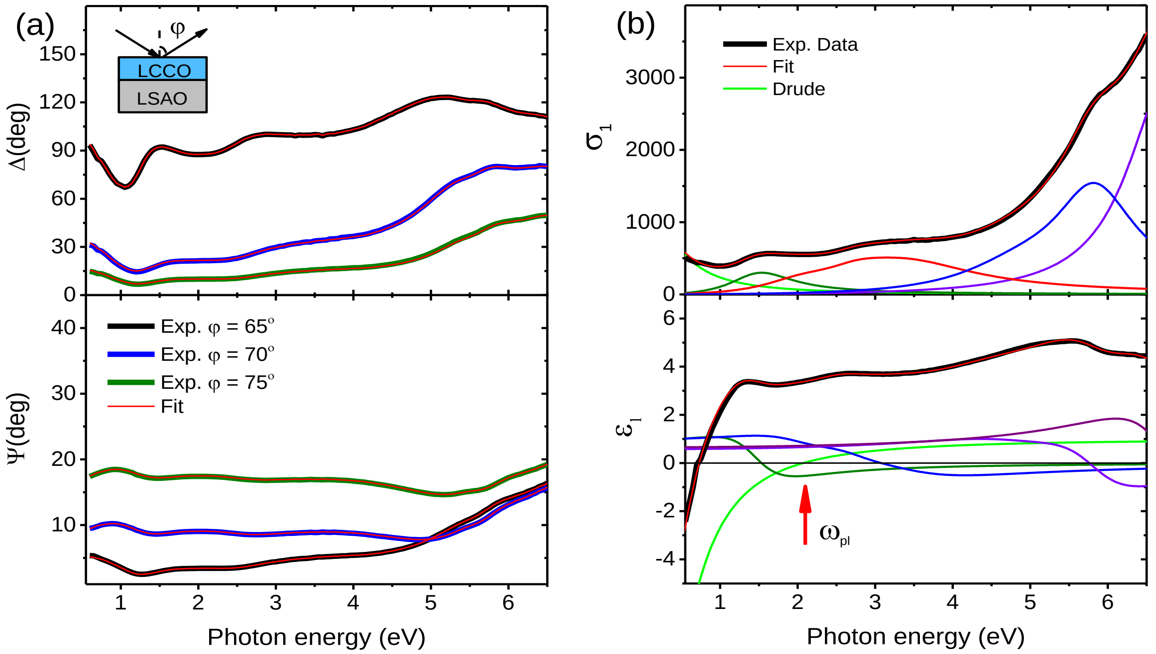

FIG. 1. (a) Fit to the ellipsometric angles $\Psi(\omega)$ and $\Delta(\omega)$ for the 10 u.c. thick $La_{1.7}Ca_{0.3}CuO_4$ film on LSAO substrate measured at angles of incidence $\varphi = 65°$ (black), $70°$ (blue), and $75°$ (green); the thin red line corresponds to the best fit using a film-on-substrate model. (b) Corresponding spectra $\sigma_1(\omega)$ and $\varepsilon_1(\omega)$ (thick black lines) represented by the total contribution (thin red lines) of separate bands determined by the dispersion analysis. The green lines show the free charge carrier contribution and the red arrow marks the unscreened plasma frequency, $\omega_{pl} \approx 2.1$ eV.

I. SPECTROSCOPIC ELLIPSOMETRY

Figure 1 shows representative ellipsometric angles $\Psi(\omega)$ and $\Delta(\omega)$ and corresponding $\varepsilon(\omega)$ and $\sigma_1(\omega)$. The ellipsometric data were fitted by point-by-point regression analysis to a film-on-substrate model, as implemented in the Woollam WVASE32® data acquisition and analysis software [1].

II. IN-SITU RHEED MONITORING

All growth runs of $La_{2-x}Ca_xCuO_4$ thin films were monitored with *in situ* RHEED, which provides information regarding the presence of parasitic phases, surface roughness, and crystal quality. The diffraction patterns in Fig. 2 exhibit clear in-plane Bragg reflections without any sign of parasitic phases for a wide range of the Ca concentration. The long and narrow

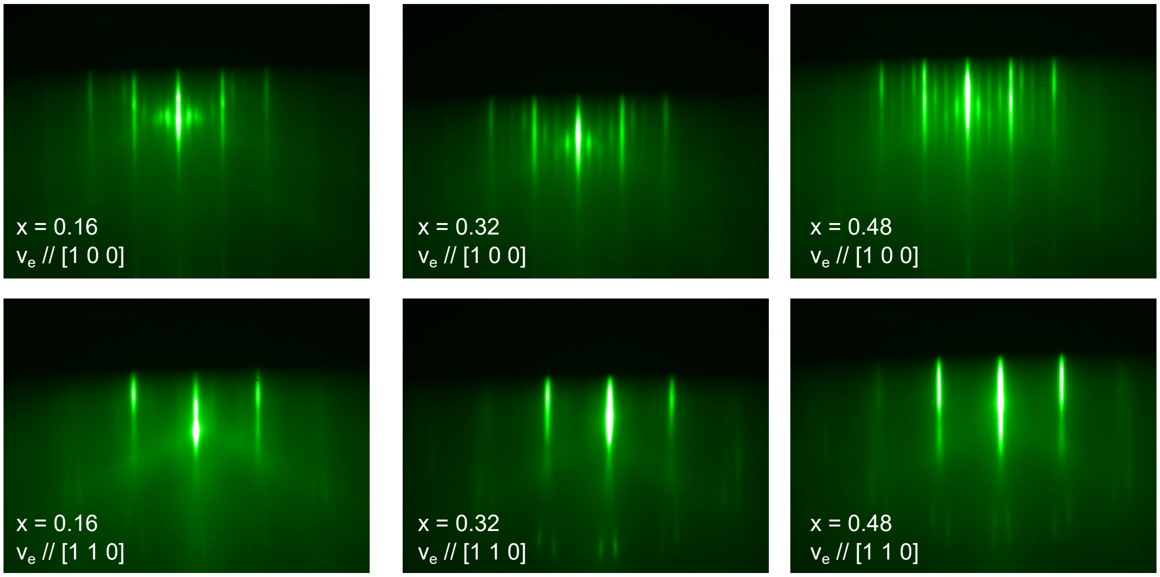

FIG. 2. *In situ* RHEED patterns of $La_{2-x}Ca_xCuO_4$ thin films. x indicates the concentration of dopants, and the [hkl] vector indicates the direction of the incoming electron beam.

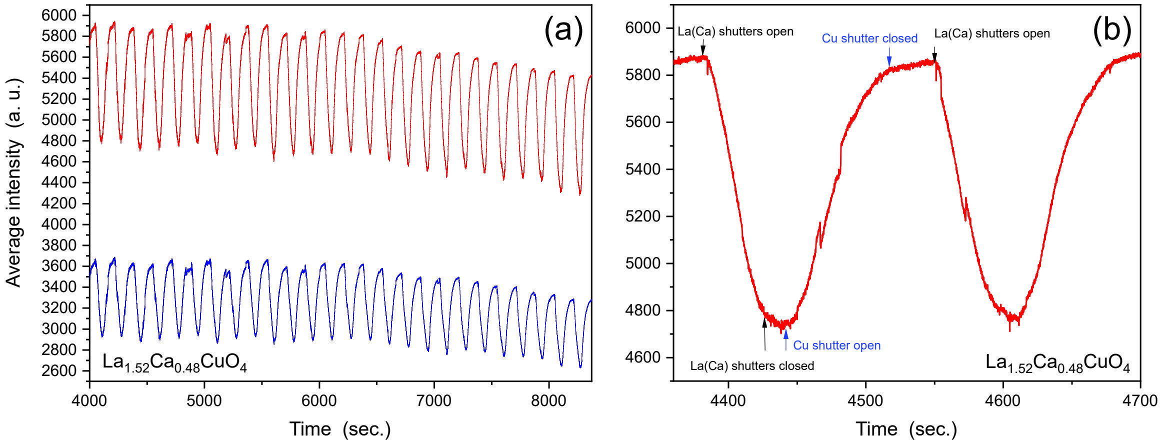

FIG. 3. (a) *In situ* RHEED intensity oscillation during MBE growth of the LCCO (x = 0.48) thin film. The red and blue curves correspond to the specular reflection and the first Bragg reflection, respectively, in the v_e// [1 0 0] configuration. (b) A magnified view of the RHEED oscillation. Arrows and texts specify the operations during sample growth.

shape of the patterns suggests smooth surfaces and high crystallinity of the films examined. In addition, the superstructure reflections in between main Bragg peaks in the [1 0 0] direction serve as complementary evidence for excellent crystallinity.

Monitoring intensity oscillation of the RHEED pattern, which contains information re-

garding coverage of the topmost layer, is an indispensable tool for MBE. We present the RHEED oscillation of LCCO(x=0.48) in Fig. 3(a), which exhibits a clear periodic modulation suggesting a stable layer-by-layer growth. The amplitude of the RHEED oscillation stays constant over the course of more than 20 repetitions, indicating that the thin film maintains its quality throughout the process. In Fig. 3(b), a magnified view of the RHEED oscillation is shown. We adjusted the shutter time of effusion cells such that the maximum intensity coincides with the end of copper deposition.

In addition to *in situ* RHEED, *ex situ* atomic force microscopy (AFM) can evaluate the roughness of thin film surfaces. We observed clear atomic-scale steps and terraces imprinted on the film by the surface structure of the substrate (Fig. 4), which suggests a smooth surface of our film. The average roughness of the measured area is $\sim$ 1 nm, which is consistent with the smooth topography and with the sharp RHEED patterns.

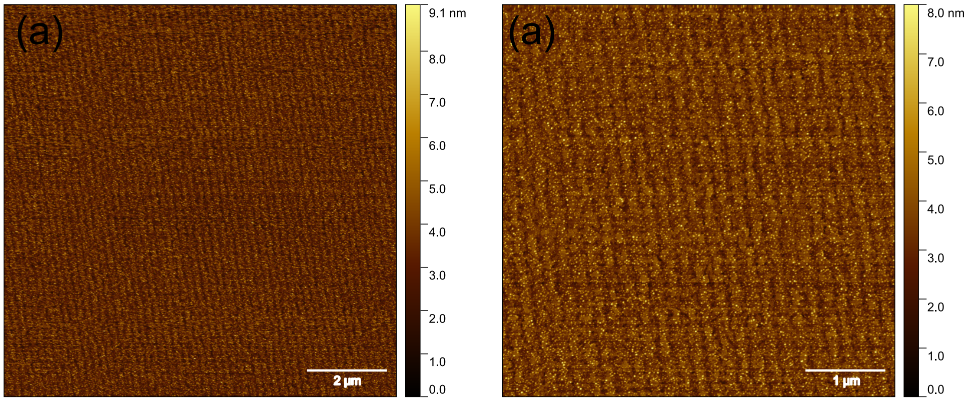

FIG. 4. (a) AFM topography image of a LCCO(x=0.4) thin film from an area of 10 μm $\times$ 10 μm. (b) AFM topography image of the sample from an area of 5 μm $\times$ 5 μm.

III. X-RAY DIFFRACTION

We carried out $\theta-2\theta$ scans along (00L) direction in order to determine c-lattice parameters and to rule out possible parasitic phases in our films. The (00L) scans of 100 u.c.-thick LCCO (x = 0.16, 0.32, 0.48) films (Fig. 5(a)) exhibit clear (00L) reflections along with (00L) reflections from the LSAO substrate suggesting that the films are well aligned with the crystal structure of the substrate. Even in thick films, no additional peak was observed confirming that our films are single-phased. The (00L) scans from 10 u.c.-thick LCCO (0.05 $\leq$ x $\leq$ 0.5) films are presented in Fig. 5(b), and they exhibit no sign of parasitic phases.

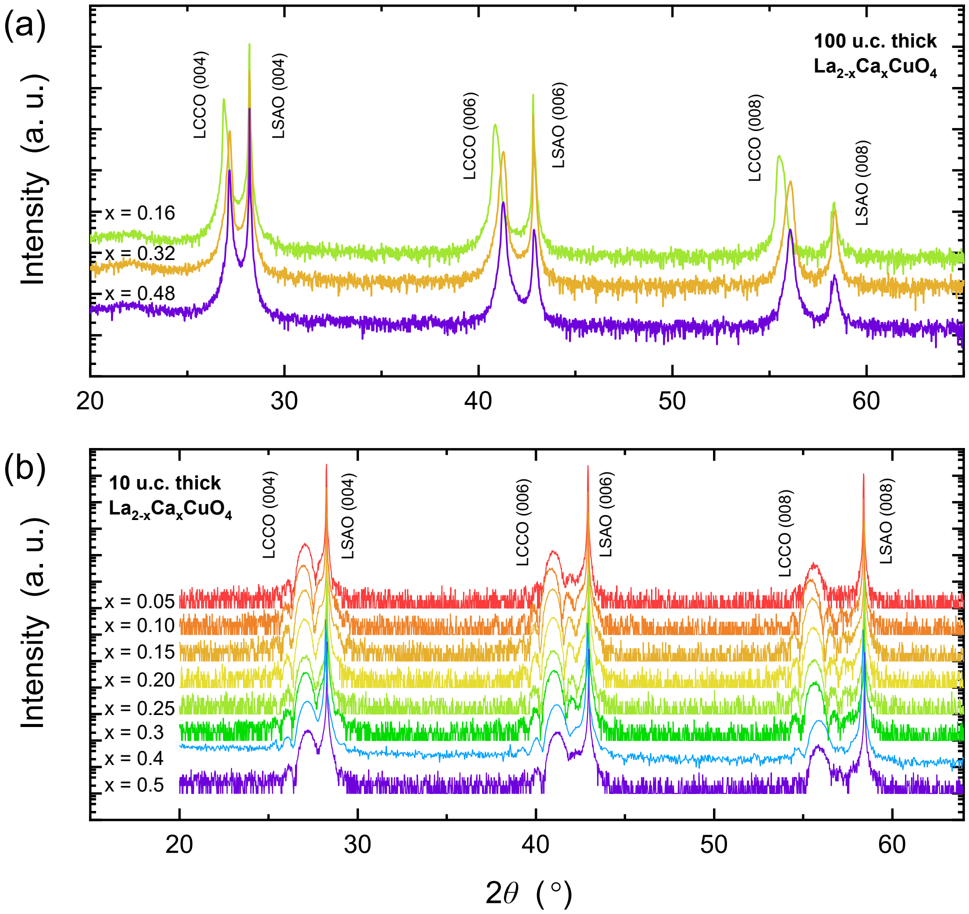

FIG. 5. wide-range (00L) XRD curves of (a) 100 unit-cells thick films, and (b) 10 unit-cells thick films.

Above x = 0.5, XRD (006) peaks become broader, and Laue fringes disappear, which indicates that the sample suffers from decomposition due to the solubility limit (see Fig. 6(a)). Because of the proximity to the solubility limit, the sample synthesis was challenging.

To exclude the samples that are decomposed, we used the c-lattice parameters from XRD θ-2θ scans. Samples that deviated from the systematic trend were excluded from the analysis (Fig. 6(b)). Peak positions in XRD θ-2θ scans suggest that the c-lattice parameter sharply increases again to 13.17 Å after $x = 0.5$ and saturates implying the decomposition into LCCO with $x \sim 0.3$ and Ca-rich parasitic phases due to the excessive amount of chemical substitution.

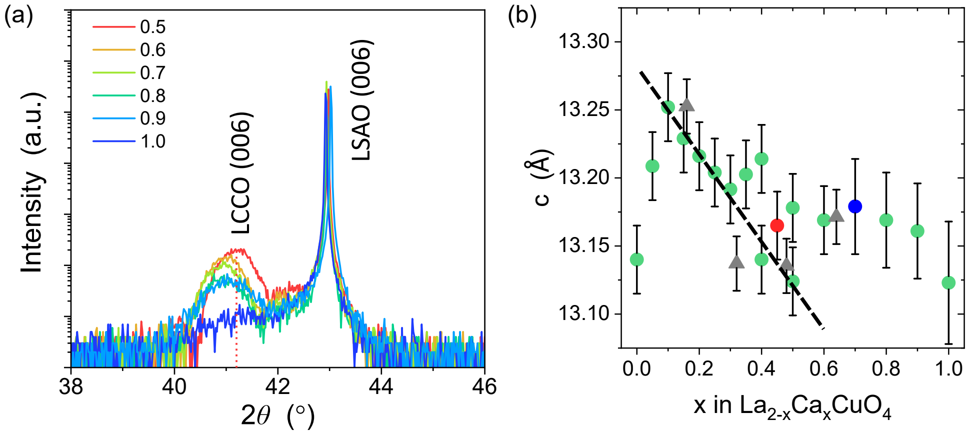

FIG. 6. XRD measurements on defective LCCO thin films with an excessive amount of Ca. (a) XRD θ-2θ scans, showing (006) reflections of LCCO thin films and LSAO substrates. (b) c-lattice parameters were calculated from XRD (00L) scans. The dashed line is a guide to the eye. Red and blue data points correspond to Fig. 1c in the main text and Fig. 3 of supplementary information, respectively.

IV. STOICHIOMETRY ASSESMENT WITH STEM AND RBS

For scanning transmission electron microscopy (STEM) analysis a probe-aberration-corrected JEOL JEM-ARM200F STEM equipped with a cold field-emission electron source, a probe Cs-corrector (DCOR, CEOS GmbH), a Gatan GIF Quantum ERS spectrometer with a K2 direct electron detector, and a large solid-angle JEOL Centurio SDD-type energy-dispersive x-ray spectroscopy (EDXS) detector were used at 200 kV. STEM imaging and EELS, EDXS analyses were performed at probe semi-convergence angles of 20 and 28 mrad, resulting in probe sizes of 0,8 and 1.0 Å, respectively. A collection semi-angle of 111 mrad and a 0.5 eV/ch dispersion with an effective energy resolution of $\sim$1 eV were used for EELS

investigations.

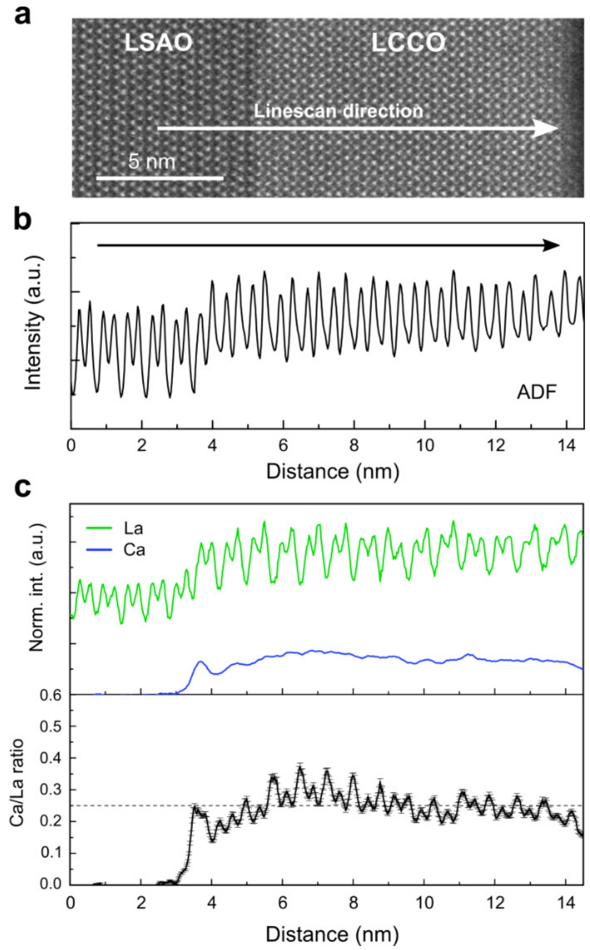

FIG. 7. (a) Cross-sectional STEM-HAADF image of a Ca-doped LCO (x=0.4) film on a LSAO (001) substrate, showing absence of crystallographic defects. (b) ADF intensity profile along the white arrow in (a). (c) Intensity profiles of La in green, Ca in blue (upper panel) and Ca/La ratio (lower panel), showing homogeneous dopant distribution with an averaged Ca/La ratio of ~0.25.

In order to demonstrate the homogeneous distribution of dopants, we carried out element-sensitive analyses via both STEM-electron energy loss spectroscopy (STEM-EELS) and STEM-energy-dispersive x-ray spectroscopy(STEM-EDXS). For STEM analyses we chose a 10 u.c.-thick LCCO (x=0.4) film. Before detailed analyses of elemental distribution, a STEM-HAADF image across the interface between the film and the substrate was obtained as presented in Fig. 7(a). The image of the representative region exhibits a coherent interface and an excellent structure of the film. Further insight was obtained using the line profile of the image (Fig. 7(b)), where the highly regular oscillatory behavior supports uniform

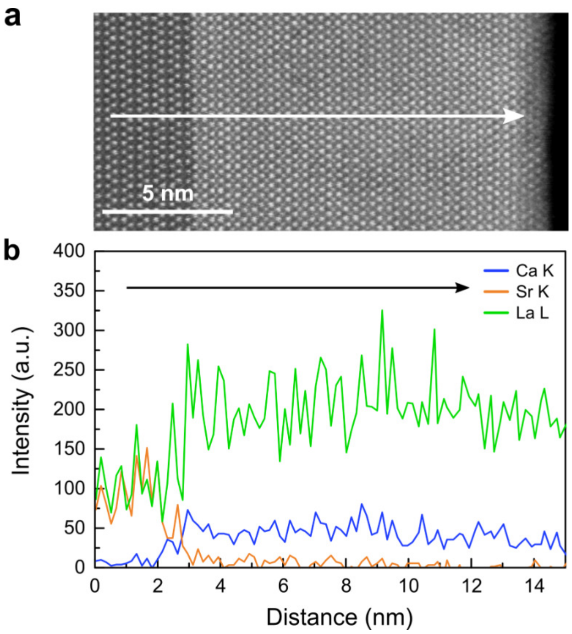

FIG. 8. (a) Cross-sectional STEM-HAADF image of a Ca-doped LCO (x=0.4) film on a LSAO (001) substrate. (b) Intensity profile of STEM EDXS along the white arrow in (a), demonstrating homogeneous distribution of Ca.

structural quality.

Figure 7(c) shows the STEM-EELS analysis focusing on the concentration of La and Ca. The intensity profiles of La and Ca are stable except for small deviation at the interface. The ratio between La and Ca measured by STEM-EELS matches the intended value from the MBE growth, Ca/La = 0.25, and it proves our good control over composition and uniform dopant-distribution.

STEM-EDXS is another method to obtain the information regarding atomic-scale stoichiometry. The STEM-EDXS line profile was collected from the region specified in Fig. 8(a). The line profiles show a homogeneous elemental distribution, consistent with the result of STEM-EELS measurement. An additional piece of information from STEM-EDX is the concentration of Sr, which reveals the width of the interface, because Sr comes exclusively from the substrate. Indeed, the concentration of Sr rapidly decays and reaches zero, and the concentrations of La and Ca stay constant after the Sr concentration reaches zero.

A complementary method to obtain the average composition of a thin film is Rutherford Backscattering Spectroscopy (RBS). Here, we used a 10 u.c.-thick LCCO (x=0.5) thin film on a MgO (100) substrate. MgO was chosen instead of LSAO in order to avoid the overlap

between elements in our film and the LSAO substrate. From the measurement we obtained a Ca/La ratio of 0.322, which coincides with the intended ratio of 0.333.

V. SAMPLES BEYOND THE SOLUBILITY LIMIT

In order to investigate decomposed samples that deviate from the systematic trend, we acquired a STEM image on the heavily doped samples with nominal $x = 0.7$ (Fig. 9). The sample with $x = 0.7$ shows extended defects that are dark in the HAADF image. These dark features in the HAADF image come from light elements that have a lower scattering cross-section with the electron beam, which is in this case Ca. Therefore, we could conclude that LCCO reached the solubility limit at $x \approx 0.5$.

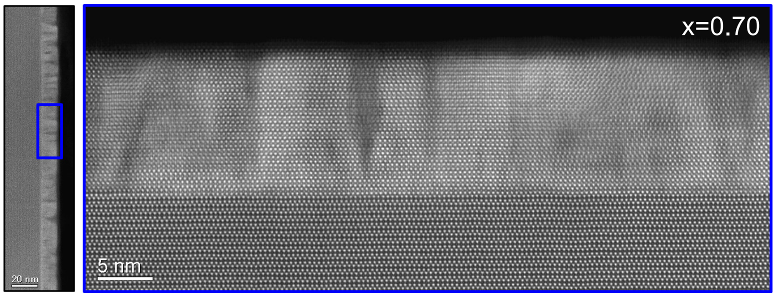

FIG. 9. STEM measurement of LCCO with x=0.7. The blue rectangle indicates the area on the right panel.

In addition to the collapsing XRD curves of decomposed samples, we found another clear sign of decomposition from optical spectroscopy. The systematic evolution of optical spectra in the main text evidenced the formation of a homogeneous solid solution, nevertheless optical spectra of samples with $x \geq 0.6$ exhibit an erratic trend showing a sudden suppression in σ_1 in the wide range of energy instead of the spectral weight transfer shown by samples with lower x (see Fig. 10). This again suggests decomposition for samples exceeding the solubility limit. Such a suppression has previously been reported in optical studies of non-superconducting overdoped bulk LSCO samples [2].

[1] J. A. Woollam Co., *Guide to using WVASE32®: Spectroscopic ellipsometry data acquisition and analysis software* (J.A. Woollam Co., Inc., Lincoln, Nebraska, 2012).

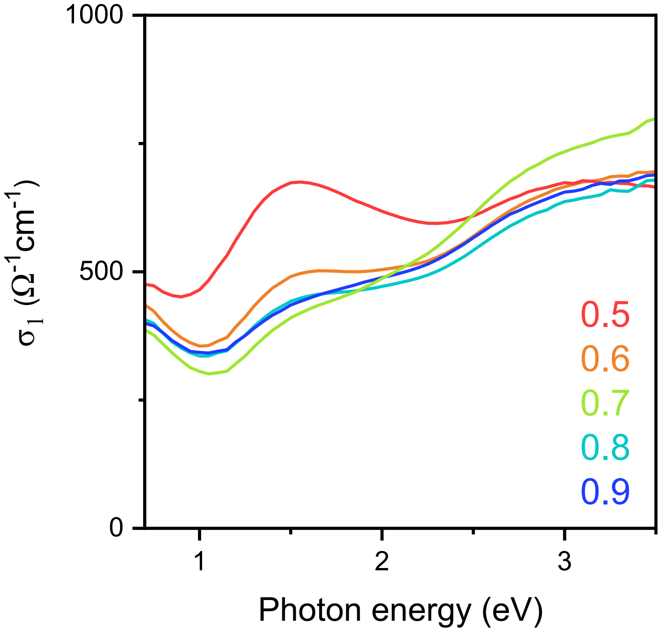

FIG. 10. Optical conductivity, σ_1, of the samples with excessive amount of dopants.

[2] S. Uchida, T. Ido, H. Takagi, T. Arima, Y. Tokura, and S. Tajima, "Optical spectra of $La_{2-x}Sr_xCuO_4$: Effect of carrier doping on the electronic structure of the CuO_2 plane," Phys. Rev. B **43**, 7942–7954 (1991).
